# Transcriptome Profiling Provides Insight into the Genes in Carotenoid Biosynthesis during the Mesocarp and Seed Developmental Stages of Avocado (*Persea americana*)

**DOI:** 10.3390/ijms20174117

**Published:** 2019-08-23

**Authors:** Yu Ge, Zhihao Cheng, Xiongyuan Si, Weihong Ma, Lin Tan, Xiaoping Zang, Bin Wu, Zining Xu, Nan Wang, Zhaoxi Zhou, Xinge Lin, Xiangshu Dong, Rulin Zhan

**Affiliations:** 1 Haikou Experimental Station, Chinese Academy of Tropical Agricultural Sciences, Haikou 570102, China; 2 Biotechnology Center, Anhui Agricultural University, Hefei 230036, China; 3 College of Agriculture, Yunnan University, Kunming 650091, China

**Keywords:** avocado, carotenoid biosynthesis, mesocarp, seed, de novo assembly from short read sequencing, full-length transcript sequencing, differentially expressed genes, gene dosage

## Abstract

Avocado (*Persea americana* Mill.) is an economically important crop because of its high nutritional value. However, the absence of a sequenced avocado reference genome has hindered investigations of secondary metabolism. For next-generation high-throughput transcriptome sequencing, we obtained 365,615,152 and 348,623,402 clean reads as well as 109.13 and 104.10 Gb of sequencing data for avocado mesocarp and seed, respectively, during five developmental stages. High-quality reads were assembled into 100,837 unigenes with an average length of 847.40 bp (N50 = 1725 bp). Additionally, 16,903 differentially expressed genes (DEGs) were detected, 17 of which were related to carotenoid biosynthesis. The expression levels of most of these 17 DEGs were higher in the mesocarp than in the seed during five developmental stages. In this study, the avocado mesocarp and seed transcriptome were also sequenced using single-molecule long-read sequencing to acquired 25.79 and 17.67 Gb clean data, respectively. We identified 233,014 and 238,219 consensus isoforms in avocado mesocarp and seed, respectively. Furthermore, 104 and 59 isoforms were found to correspond to the putative 11 carotenoid biosynthetic-related genes in the avocado mesocarp and seed, respectively. The isoform numbers of 10 out of the putative 11 genes involved in the carotenoid biosynthetic pathway were higher in the mesocarp than those in the seed. Besides, alpha- and beta-carotene contents in the avocado mesocarp and seed during five developmental stages were also measured, and they were higher in the mesocarp than in the seed, which validated the results of transcriptome profiling. Gene expression changes and the associated variations in gene dosage could influence carotenoid biosynthesis. These results will help to further elucidate carotenoid biosynthesis in avocado.

## 1. Introduction

Avocado (*Persea americana* Mill.) is a member of the family Lauraceae of the order Laurales, and widely grown in countries and regions with a tropical-to-cool climate [1,2,3]. Avocado is among the most economically important subtropical/tropical fruit crops worldwide, with considerable increases in yield reported in several countries, including Mexico, the USA, Indonesia, Chile, Spain, Israel, Colombia, South Africa, and Australia [4]. Certain avocado constituents, such as carotenoids, lipids, sugars, proteins, minerals, vitamins, and other nutrients and active ingredients, provide nutritional and health benefits [5,6,7].

Carotenoids in fruits have been extensively studied because of their nutritional benefits for humans [5,8]. Moreover, some carotenoids serve as precursors of vitamin A, strigolactones, and abscisic acid, and some apocarotenoids are also potent antioxidants and colorants [9]. Additionally, carotenoid derivatives substantially affect the aromatic flavor of fruits, thereby making the fruits more desirable to consumers and seed dispersers [10,11]. Carotenoids are natural yellow-to-red pigments that are mainly a type of C40 terpenoid distributed in most photosynthetic organisms as well as in some non-photosynthetic fungi and bacteria [12]. In plants, carotenoids primarily contribute to photosynthesis, photomorphogenesis, photoprotection, light-harvesting processes, and growth and development [12,13,14]. The plant carotenoid metabolic pathway has been well elucidated. This pathway consists of a series of chemical reactions, including condensations, dehydrogenations, cyclizations, hydroxylations, and epoxidations [12].

Next-generation high-throughput sequencing technology (NGST) have recently become popular options for transcriptome sequencing experiments because they enable high-throughput, efficient, accurate, and reproducible analyses [15,16,17,18,19,20]. Previous studies suggested that changes in carotenoid biosynthesis and accumulation are correlated with changes to the expression of genes encoding carotenoid metabolic pathway enzymes [8,12]. Transcriptome sequencing methods have been used to investigate the carotenoid biosynthetic mechanism in many species, including *Momordica cochinchinensis* [21], *Brassica campestris* [22], green alga [23], celery [9], carrot [24,25], and *Euscaphi skonishii* [26]. However, few reports have focused on transcriptome sequencing to investigate the carotenoid biosynthetic mechanism in avocado.

Among the third-generation sequencing platforms, PacBio RS II, which is regarded as the first commercialized third-generation sequencer, is based on single-molecule real-time (SMRT) technology [27]. The PacBio RS II system can produce much longer reads than second-generation sequencing platforms, and has been applied to effectively capture full-length transcript sequences [28]. Single-molecule real-time technology has the following three main advantages over second-generation sequencing options: it generates longer reads, it has a higher consensus accuracy, and it is less biased [29]. A previous study revealed that SMRT technology can precisely ascertain alternative polyadenylation sites and full-length splice isoforms, and also detect a higher isoform density than that for the reference genome [30]. The application of SMRT technology for nearly 3 years has helped to elucidate the complexity of the transcriptome and molecular mechanism underlying metabolite synthesis in safflower [27], *Zanthoxylum bungeanum* [28], *Trifolium pretense* [30], sugarcane [31], switchgrass [32], *Medicago sativa* [33], *Zanthoxylum planispinum* [34], bermudagrass [35], *Camellia sinensis* [36], and *Cassia obtusifolia* [37]. So far, no report has been found about the application of SMRT technology in a plant species from the family Lauraceae.

In this study, Illumina HiSeq 2000 next-generation sequencing technology and PacBio RS II third-generation sequencing platform were integrated to generate transcriptome data for exploring the carotenoid biosynthetic pathways in the avocado mesocarp and seed. We integrated the gene dosage variation and the associated changes in gene expression to identify genes that are likely important for carotenoid accumulation. Additionally, metabolite profiling (alpha- and beta-carotene contents) via high-performance liquid chromatography (HPLC) was used to auxiliarily validate the transcriptomic analyses. The data obtained in this comprehensive study involving the full-length transcript sequences and de novo transcriptome assembly from short read sequencing will be useful for investigating the main physiological and biochemical molecular metabolic mechanisms in the avocado mesocarp and seed.

## 2. Results

### 2.1. Overview of the Morphology and NGST Profiling

Morphological measurements, including mesocarp weight, seed weight, fruit length, and fruit width, gradually increased from 75 to 215 days after full bloom (DAFB), peaking at 114.74 g FW, 11.81 g FW, 83.07 mm, and 62.42 mm, respectively (Table S1). The photos of the tested avocado samples per fruit developmental stage are also presented in Figure S1. The RNA extracted from 15 mesocarp and seed samples were analyzed by RNA sequencing (RNA-seq), with three replicates per avocado fruit developmental stage. The sequencing of 30 cDNA libraries resulted in 20–26 million clean reads and 6.03–8.28 Gb of sequence data (Table S2). The generated avocado transcriptome data were deposited in the GenBank database (accession number PRJNA541745). The default parameters of the Trinity program were used to assemble the high-quality reads into 205,415 transcripts with a mean length of 1199.57 bp (N50 = 2063 bp) as well as 100,837 unigenes with a mean length of 847.40 bp (N50 = 1725 bp). Of these 100,837 unigenes, 59,969 (59.47%) were short (i.e., up to 500 bp), 16,511 (16.37%) were 501–1000 bp long, and 24,357 (24.16%) were longer than 1000 bp. The length distributions of all transcripts and unigenes are presented in Figure S2. These results demonstrated that the sequencing quality was sufficient for subsequent analyses.

### 2.2. Annotation and Identification of Unigenes

**Figure 1:**
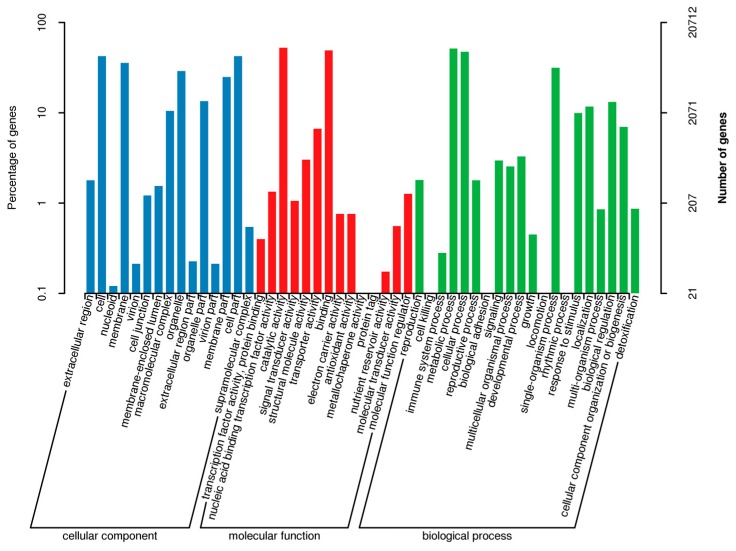
Gene Ontology functional annotations of avocado unigenes. The unigenes were divided into three categories (biological process, cellular component, and molecular function). A total of 19,065 unigenes were functionally characterized based on 26 Eukaryotic Orthologous Groups (KOG) categories (Figure 2). Among the 26 categories, ‘General function prediction only’ represented the largest group (5557 unigenes, 26.38%), followed by ‘Posttranslational modification, protein turnover, chaperones’ (1959 unigenes, 9.30%), and ‘Signal transduction mechanisms’ (1729 unigenes, 8.19%). ‘Extracellular structures’ and ‘Cell motility’ were the two smallest groups. Additionally, 12,638 unigenes were assigned to 130 pathways in the Kyoto Encyclopedia of Genes and Genomes (KEGG) database (Table S4), with carbon metabolism (571 unigenes), biosynthesis of amino acids (516 unigenes), and ribosome (465 unigenes) representing the three most common pathways.

Regarding the gene annotations, the BLASTX program revealed that 14,565 (37.05%), 20,712 (52.69%), 12,638 (32.15%), 19,065 (48.50%), 23,009 (58.54%), 22,403 (56.99%), 34,394 (87.50%), and 35,021 (89.09%) of the 39,309 avocado unigenes had significant matches with sequences in the Clusters of Orthologous Groups (COG), Gene Ontology (GO), Kyoto Encyclopedia of Genes and Genomes (KEGG), Eukaryotic Orthologous Groups (KOG), Pfam, Swiss-Prot, eggNOG, and the NCBI non-redundant protein sequence (Nr) databases, respectively. To further predict and classify functions, the annotated unigenes were analyzed according to GO assignments, COG classifications, and KEGG pathway assignments. A total of 20,712 unigenes were assigned to 49 sub-categories of the three main GO functional categories (cellular component, biological process, and molecular function) (Figure 1; Table S3). The molecular function category comprised the most unigenes, followed by the biological process and cellular component categories. The most common molecular function GO terms were catalytic activity (10,855 unigenes, GO: 0003824) and binding (10,148 unigenes, GO: 0005488). The top three biological process GO terms were metabolic process (10,654 unigenes, GO: 0008152), cellular process (9833 unigenes, GO: 0009987), and single-organism process (6516 unigenes, GO: 0044699). The most frequently observed cellular component GO terms were cell (8781 unigenes, GO: 0005623) and cell part (8769 unigenes, GO: 0044464) (Figure 1).

### 2.3. Screening of Differentially Expressed Genes during Avocado Mesocarp and Seed Development

An analysis of differentially expressed genes (DEGs) in the avocado mesocarp and seed during five fruit developmental stages revealed 16,903 DEGs (Table S5). There were 4013–4828 DEGs between the mesocarp and seed at five time-points, with some minor variability in the number of DEGs among the five fruit developmental stages. The number of DEGs increased considerably during mesocarp development. The largest number of DEGs (1516) was detected between 75 and 215 DAFB. Similarly, the number of DEGs sharply increased during the whole seed development stage, with a 4.40-fold increase from 75 vs. 110 DAFB to 75 vs. 215 DAFB. These results indicated that the fifth mesocarp and seed developmental stages may be associated with the most dramatic changes in enzyme contents and multiple metabolic pathways.

### 2.4. Differentially Expressed Carotenoid Biosynthetic Genes between the Avocado Mesocarp and Seed

**Figure 3:**
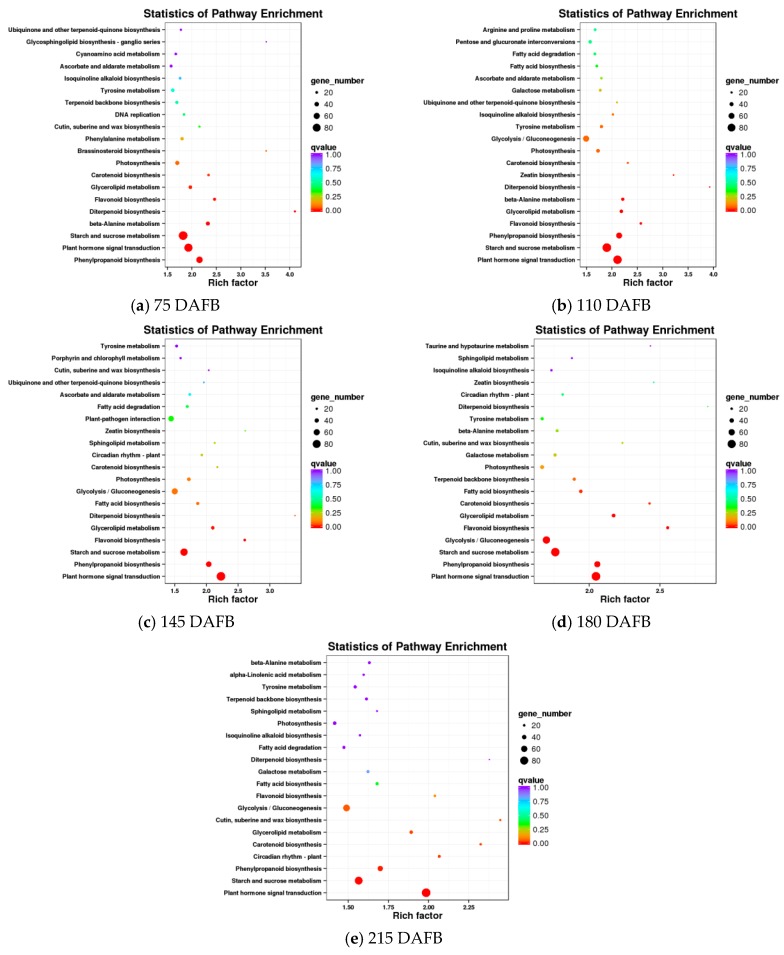
Results of the KEGG enrichment analysis of differentially expressed genes (DEGs) between the avocado mesocarp and seed at five developmental stages.

A comparison of the avocado mesocarp and seed at five developmental stages based on the KEGG pathway enrichment among all DEGs resulted in the identification of the carotenoid biosynthetic pathway in four of the five developmental stages (Figure 3). The DEGs detected in the avocado mesocarp and seed transcriptomes included 17 unigenes that putatively encode 11 enzymes in the carotenoid biosynthetic pathway (Table 1). 

**Figure 4:**
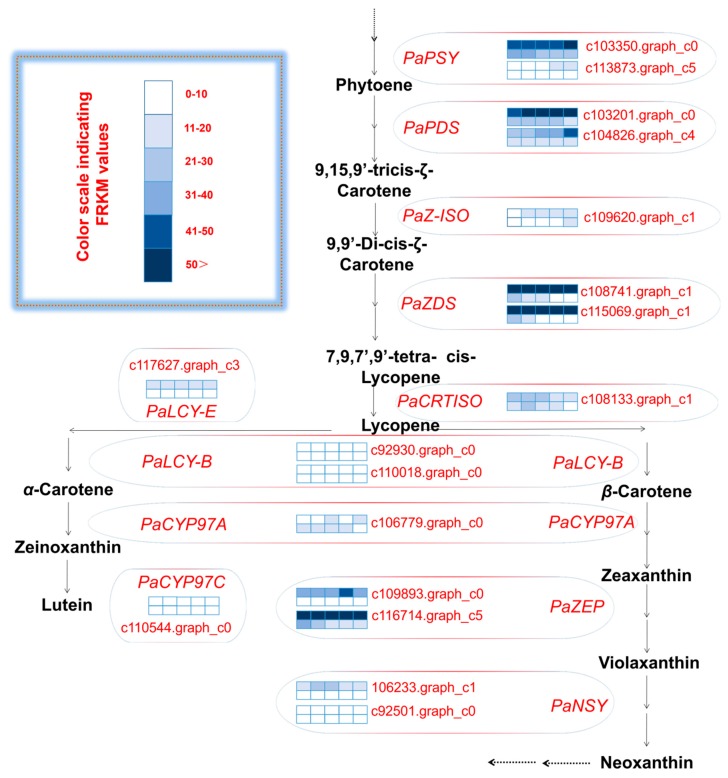
Carotenoid biosynthetic pathway and transcript levels during avocado mesocarp and seed developmental stages. FPKM: fragments per kilobase of transcript sequence per million base pairs sequenced; gene expression levels at 75, 110, 145, 180, and 215 days after full bloom (DAFB) are indicated with colored bars; the top and bottom barfor eachunigene demonstrates avocado mosocarp and seed, respectively; PSY: 15-cis-phytoene synthase; PDS: phytoene desaturase; Z-ISO: 15-cis-*ζ*-carotene isomerase; ZDS: *ζ*-carotene desaturase; CRTISO: carotenoid isomerase; LCY-E: lycopene *ε*-cyclase; LCY-B: lycopene *β*-cyclase; CYP97A: P450 *β*-ring carotene hydroxylase; CYP97C: P450 *ε*-ring carotene hydroxylase; ZEP: zeaxanthin epoxidase; NSY: neoxanthin synthase.

**Figure 5:**
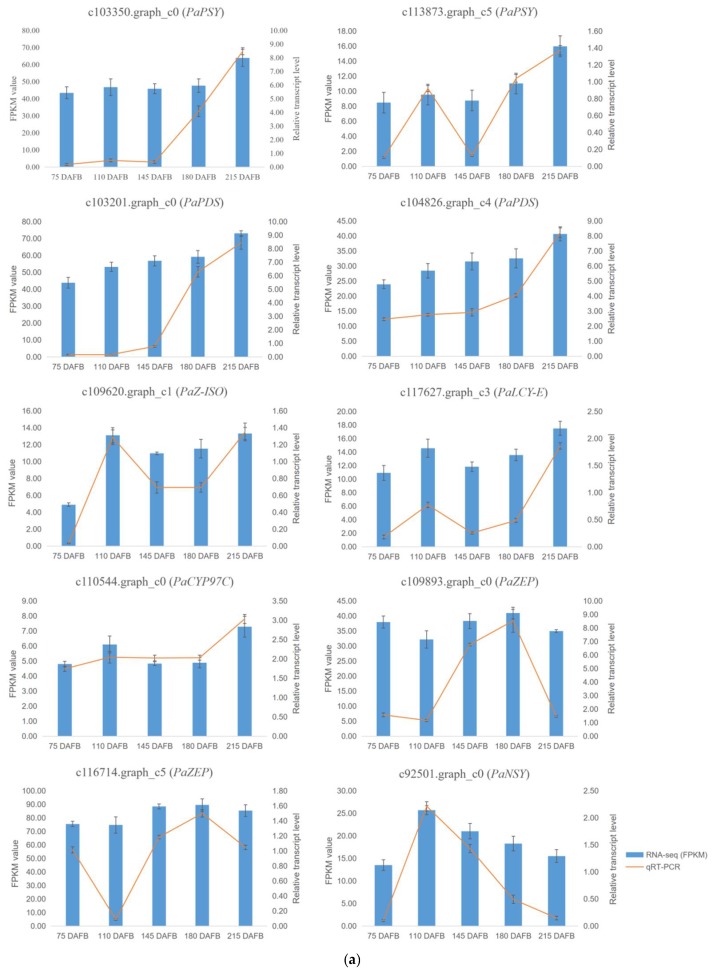

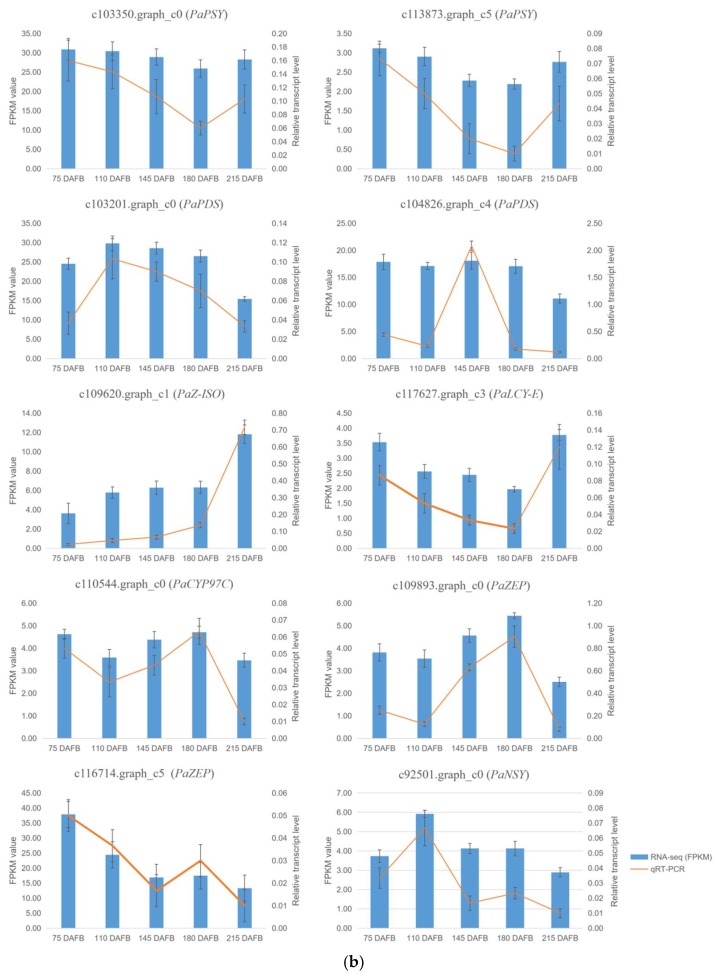
The FPKM values and relative expression levels of 10 carotenoid biosynthesis unigenes in avocado mesocarp (**a**) and seed (**b**). Error bars are standard errors of the mean from three biological replicates and two technical replicates.

An analysis of the unigenes related to carotenoid biosynthesis that were differentially expressed during five mesocarp and seed developmental stages (Figure 4) revealed that the following 15 unigenes were more highly expressed in the mesocarp than in the seed at each of the five examined time-points: *PaPSY* (c103350.graph_c0 and c113873.graph_c5), *PaPDS* (c103201.graph_c0 and c104826.graph_c4), *PaZ-ISO* (c109620.graph_c1), *PaZDS* (c108741.graph_c1 and c115069.graph_c3), *PaCRTISO* (c108133.graph_c1), *PaLCY-E* (c117627.graph_c3), *PaLCY-B* (c92930.graph_c0 and c110018.graph_c0), *PaCYP97C* (c110544.graph_c0), *PaZEP* (c109893.graph_c0 and c116714.graph_c5), and *PaNSY* (c92501.graph_c0). In contrast, the *PaNSY* (c106233.graph_c1) expression level was considerably lower in the mesocarp than in the seed at each of the five time-points (Figure 4; Table S6). Additionally, *PaCYP97A* (c106779.graph_c0) was expressed at lower levels in the mesocarp than in the seed from 75 to 180 DAFB, but the opposite pattern was observed at 215 DAFB (Figure 4; Table S6). The *PaPSY*, *PaPDS*, *PaZ-ISO*, *PaZDS*, *PaCRTISO*, *PaLCY-E*, and *PaLCY-B* expression levels were higher in the mesocarp than in the seed at each of the five time-points, and increased by 1.09 to 22.41 fold (Table S6). To confirm the accuracy of the high-throughput sequencing results, the expression levels of ten unigenes involved in the carotenoid biosynthetic pathway (i.e., *PaPSY*, *PaPDS*, *PaZ-ISO*, *PaLCY-E*, *PaCYP97C*, *PaZEP*, and *PaNSY*) were analyzed by a quantitative real-time polymerase chain reaction (qRT-PCR) assay (Figure 5). The resulting expression patterns of these genes during the five mesocarp and seed developmental stages were consistent with the RNA-seq data.

### 2.5. General Properties of Single-Molecule Long-Reads

Full-length cDNA sequences derived from poly-A-tailed RNA samples were normalized and subjected to SMRT sequencing with the PacBio RS II platform. A total of 25.79 and 17.67 Gb clean data were generated for the library in avocado mesocarp and seed, respectively. Each SMRT cell produced 651,260 and 586,430 reads of inserts (ROIs) from the library (1–6 kb) in avocado mesocarp and seed, respectively. These ROIs were successfully extracted in avocado mesocarp and seed, respectively, with a mean length of 2200 and 2239 bp, a quality score of 0.96 and 0.94. All ROIs were further classified into 495,245 and 403,108 full-length nonchimeric in avocado mesocarp and seed, respectively. On the basis of the iterative isoform-clustering algorithm, 233,014 and 238,219consensus isoforms were acquired in avocado mesocarp and seed, respectively, with a mean length of 2170 and 2027 bp (Table S7). After removing the redundant sequences for all high-quality transcripts and corrected low-quality transcripts with CD-HIT (c = 0.90), 76,345 and 68,618 nonredundant transcripts remained. The SMRT and Illumina HiSeq 2000 sequencing data were deposited in the GenBank database (accession numbers PRJNA551932 and PRJNA559779).

### 2.6. Isoforms in Carotenoid Biosynthetic Pathway between the Avocado Mesocarp and Seed

**Figure 6:**
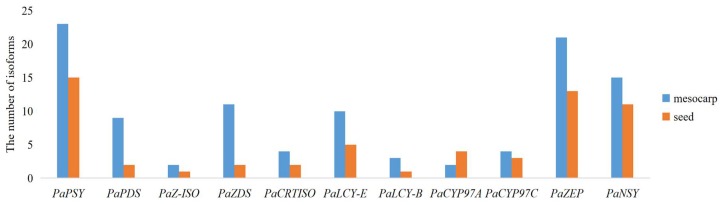
Number of single-molecule real-time (SMRT) isoforms corresponding to the putative 11 genes in the carotenoid biosynthetic pathway.

KEGG analysis in the avocado mesocarp and seed indicated that a total of 104 and 59 isoforms were found to correspond to the putative 11 genes in the carotenoid biosynthetic pathway, respectively (Figure 6). Two to 23 isoforms were found in the putative 11 genes in avocado mesocarp, and one to 15 isoforms were generated from the putative 11 genes in avocado seed. *PaPSY* possessed the most isoform number in avocado mesocarp and seed, respectively. The number of isoforms correspond to the putative 10 genes in the carotenoid biosynthetic pathway were higher in the mesocarp than those in the seed, and increased by 1.33–5.50 fold. However, the number of isoforms corresponding to *PaCYP97A* was lower in the mesocarp than those in the seed.

### 2.7. Verification of Transcriptome Profiling in Carotenoid Biosynthetic Pathway between the Avocado Mesocarp and Seed by Metabolite Profiling via HPLC

**Figure 7:**
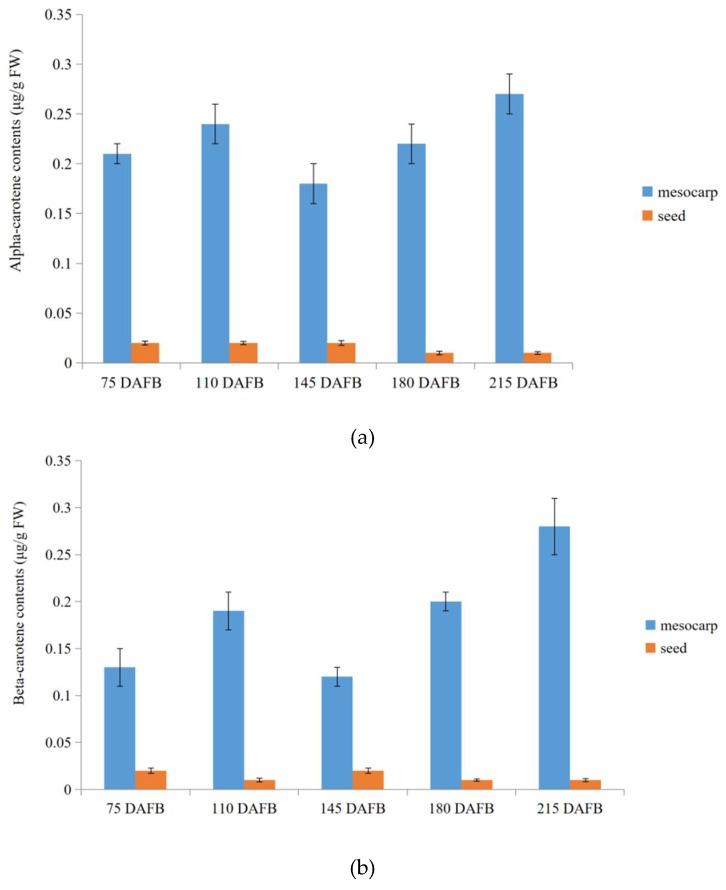
Alpha-carotene and beta-carotene contents during five avocado mesocarp (**a**) and seed (**b**) developmental stages.

At last, in order to validate transcriptome profiling via NGST and SMRT sequencing in carotenoid biosynthetic pathway between the avocado mesocarp and seed, alpha- and beta-carotene were selected to measure contents during five avocado mesocarp and seed developmental stages by HPLC (Figure S3). The mesocarp alpha- and beta-carotene contents increased slightly from 75 days after full bloom (DAFB) (0.21 and 0.13 μg/g fresh weight (FW), respectively) to 110 DAFB (0.24 and 0.19 μg/g FW, respectively). They then decreased to their lowest levels (0.18 and 0.12 μg/g FW, respectively) at 145 DAFB, but then increased again up to 210 DAFB, peaking at 0.27 and 0.28 μg/g FW, respectively (Figure 7). Trace amounts of alpha- and beta-carotenes were detected in developing seeds, with the contents fluctuating between 0.01 and 0.02 μg/g FW from 75 to 215 DAFB (Figure 7).

## 3. Discussion

As it is inexpensive and can be completed rapidly, the transcriptome sequencing technique is useful for obtaining a large number of unigene sequences for an organism that lacks an available reference sequence [38]. To the best of our knowledge, for avocado, NGST transcriptome sequencing has been used to investigate fatty acid biosynthesis [39,40,41], but not any other metabolic biosynthetic pathway. Within our transcriptome assembly, 109.13 and 104.10 Gb of sequence data were respectively generated for the avocado mesocarp and seed during five developmental stages. Additionally, the 100,837 identified unigenes may be useful for subsequent analyses of metabolic biosynthetic pathways in avocado or related species. The N50 and mean lengths of avocado unigenes in our study were 1725 and 847.40 bp, respectively, which implies that our sequence assembly was accurate and effective. The N50 value in this study was higher than those obtained for avocado samples generated from mesocarp during four developmental stages (1050 bp) [41] and our previous avocado samples from five mixed organs sampled (1283 bp) [42], while the mean length in this study was lower than those obtained for both studies (987 and 922 bp) [41,42]. Recently, one of the advances in transcriptome sequencing technology has been the development of the long-read SMRT sequencing technique, which enables researchers to obtain a substantial number of full-length sequences from a cDNA library [28]. In the current study, PacBio SMRT system was applied to generate the full-length transcriptome of avocado mesocarp and seed. The 25.79 and 17.67 Gb SMRT data produced in this study provide the comprehensive insights into the avocado mesocarp and seed, respectively, and might serve as the genetic basis for future research on avocado. Interestingly, the full-length transcriptome sequence described herein is also the first such sequence for a plant species from the family Lauraceae.

Carotenoids are widely distributed isoprenoid pigments with very diverse biological functions in plants [12]. Carotenoids accumulate as secondary metabolites in leaves [9,22], fruits [21,26,43], and roots [24,25]. The carotenoid biosynthetic pathway has been extensively studied in many photosynthetic and non-photosynthetic organisms, and some researchers confirmed that in most plant species, carotenoid accumulation is mainly controlled by regulating the transcription of genes related to carotenoid biosynthesis [12]. However, the transcript profiles of genes related to carotenoid biosynthesis in avocado fruit remained unclear. In our avocado NGST transcriptome database, we identified 17 unigenes encoding 11 putative enzymes involved in the carotenoid biosynthetic pathway in avocado fruit. The 15 out of 17 unigenes were more highly expressed in the mesocarp than in the seed at each of the five examined time-points. Meanwhile, SMRT transcriptome database in our study indicated that the number of isoforms correspond to the putative 10 genes in the carotenoid biosynthetic pathway were higher in the mesocarp than those in the seed. Furthermore, the metabolite (alpha- and beta-carotene) profiling via HPLC in the avocado mesocarp and seed during five developmental stages in this study validated the results of our NGST and SMRT transcriptome profiling. These results clearly showed that the upregulated expression levels of most unigenes encoding 11 putative enzymes involved in the carotenoid biosynthetic pathway might contribute to the higher carotenoid pathway flux in the avocado mesocarp than in the seed. Besides, gene dosage (isoform number) increase of most carotenoid biosynthetic-related genes could also accelerate the carotenoid accumulation. Previous studies revealed gene dosage balance impacts on agronomic traits in plants, and defined the linkage between quantitative trait and gene dosage variation [44,45,46]. Consequently, we might imply that the gene dosage variation and the associated changes in gene expression of these unigenes might be important for controlling the carotenoid contents in avocado during the mesocarp and seed developmental stage.

An earlier investigation proved that upregulated *PSY* and *PDS* expression levels are correlated with the total carotenoid content during the tomato fruit maturation stage [47]. Similarly, *PSY*, *ZDS*, *CRTISO*, and *LCY-E* might be key genes for controlling carotenoid contents in *M. cochinchinensis* ripening fruits [21]. Additionally, *LCY-B* expression contributes to the accumulation of carotenoids in papaya [48], kiwifruit [49], and citrus [50] fruits. Another study indicated that *PSY* expression is also related to the alpha- and beta-carotene as well as total carotenoid contents in red pepper fruits [51]. In *B. campestris* L. subsp. *chinensis* var. *rosularis* Tsen and Lee leaves, *LCY-E* and *ZDS* expression may be vital for carotenoid biosynthesis [22]. In celery, *PSY* and *LCY-E* expression may be important for promoting beta-carotene biosynthesis. In the potato tuber, *PSY* expression is considered to increase the beta-carotene content [52]. Welsch [53] also suggested that *PSY* expression mediates the beta-carotene accumulation in cassava roots. Thus, analyses of the differences in gene expression profiles may yield new insights into carotenoid biosynthetic mechanisms and identify diverse carotenogenic genes expressed in various developmental stages, tissues, and species as well as in response to specific treatments.

The identification of genes encoding enzymes related to the carotenoid biosynthetic pathway not only facilitates the characterization of physiological functions in higher plants, it also provides useful information relevant for metabolic engineering. On the basis of NGST and SMRT transcriptome sequencing in this study, we investigated the differences in carotenoid biosynthesis between the avocado mesocarp and seed. However, carotenoid biosynthesis involves complex biological processes regulated by many biological pathways (i.e., the MVA, MEP, and carotenoid biosynthetic pathways) and genes. The NGST and SMRT transcriptome database described herein may represent a useful resource for clarifying carotenoid biosynthesis in various avocado tissues. Additionally, to the best of our knowledge, this study is the first to integrate Illumina with PacBio SMRT sequencing platforms for investigating avocado mesocarp and seed developmental stages via transcriptome sequencing and assembly without a reference genome. We believe that the transcriptome dataset will provide a solid foundation for future functional and genomics-based analyses of avocado, and will be useful for elucidating metabolic biosynthetic mechanisms.

## 4. Materials and Methods

### 4.1. Plant Materials

Avocado fruits (cultivar ‘Hass’) were harvested from six 10-year-old trees grafted onto Zutano clonal rootstock (two trees were used as a unit for each biological replicate) from April 2018 to September 2018 at the Chinese Academy of Tropical Agricultural Sciences (Danzhou, Hainan, China: 19.52°N, 109.57°E; altitude = 200 m above sea level). In these trees, fruits that developed during the main flowering season (i.e., February 2018) were marked, after which samples were collected at five time-points (75, 110, 145, 180, and 215 DAFB) until the fruits reached physiological maturity (defined as the ability to ripen after harvest). Two sets of fruits were randomly collected for each biological replicate during each developmental stage. The first set of nine fruits was used to measure fruit, mesocarp, and seed phenotypic traits. The second set of nine fruits was used for transcript and carotenoid analyses. Fruits were quickly brought to the laboratory, after which their phenotypic traits were measured as previously described [7] or they were immediately frozen at −80 °C for transcript and carotenoid analyses.

### 4.2. NGST Sequencing

Total RNA was extracted using a Plant RNA Kit (OMEGA Bio-Tek, Norcross, GA, USA). RNA concentration was measured using NanoDrop 2000 (Thermo Scientific, Waltham, MA, USA). RNA integrity was assessed using the RNA Nano 6000 Assay Kit of the Agilent Bioanalyzer 2100 system (Agilent Technologies, Santa Clara, CA, USA).mRNA was purified from total RNA with poly-T oligo-attached magnetic beads. Samples underwent an RNA-seq analysis involving three biological replicates per sample. The fragmentation step was completed with divalent cations in the NEBNext First Strand Synthesis Reaction Buffer (5×) at an elevated temperature. First-strand cDNA was synthesized with a series of random hexamer primers and reverse transcriptase, and second-strand cDNA was subsequently produced with DNA Polymerase I and RNase H. The cDNA libraries were constructed by ligating cDNA fragments to sequencing adapters and amplifying fragments by PCR. The libraries were then sequenced with the Illumina HiSeq 2000 platform (Nanxin Bioinformatics Technology Co., Ltd., Guangzhou, China).

### 4.3. Transcriptome Assembly, Annotation, and Coding Sequence Prediction

Clean data (clean reads) were obtained by discarding reads with adapters, reads with ambiguous poly-N sequences, and low-quality reads in which more than 50% of the bases had a Q-value ≤ 20. The two read files that were independently established for the libraries/samples were used for assembling the transcriptome with the Trinity program (version 2.5.1) [54]; the min_kmer_cov was set to 2 and all other parameters were set to default values. The assembled transcripts were hierarchically clustered to unigenes through shared reads and expression by the Corset program [55].

Unigenes were functionally annotated with a BLASTX alignment algorithm (E-value threshold of 10^−5^) and the following databases: KOG/COG, Swiss-Prot (manually annotated and reviewed protein sequence database), Pfam (along with the HMMER3.0 package), Nr, and Nt (comprising non-redundant nucleotide sequences). The KEGG Automatic Annotation Server [56] was used to map these genes according to the KEGG metabolic pathway database. Rich factor = (the number of DEGs/the number of all DEGs)/(the number of all unigenes in pathways/the number of all unigenes in KEGG). Additionally, Blast2GO (version 2.5) [57] was used for the annotation of unigenes with GO terms based on the BLASTX hits against the Pfam and Nr databases, with a cut-off E-value of 10^−6^. To predict the coding sequences, the unigenes were first used to screen the Nr and Swiss-Prot databases with a BLAST algorithm, after which the open reading frame data for sequence matches were acquired directly. The coding sequences for the remaining unigenes were predicted with ESTScan (version 3.0.3) (https://sourceforge.net/projects/estscan/).

### 4.4. Identification of Differentially Expressed Genes

To identify DEGs between two samples, the gene expression levels were quantified with the FPKM method. The read counts were adjusted with the edgeR program package, with one scaling-normalized factor for each sequenced library. The DEGs between two samples were analyzed with the DEGSeq R package (version 1.20.0). The *p*-values were adjusted according to the Benjamini and Hochberg method. A corrected *p*-value of 0.005 and a log_2_ (fold-change) of 1 were set as the threshold for identifying significant DEGs. Significantly enriched GO terms and KEGG pathways were determined based on a corrected *p*-value ≤ 0.05. The GO functional enrichment and KEGG pathway enrichment analyses of the DEGs were completed with GOseq R packages and KOBAS (version 2.0) (http://kobas.cbi.pku.edu.cn/home.do), respectively.

### 4.5. Validation of Transcripts by Quantitative Real-Time PCR

The expression levels of 10 unigenes related to carotenoid biosynthesis in the avocado mesocarp and seed were validated by a qRT-PCR assay, which was completed with a 96-well plate and the QuantStudio 7 Flex Real Time PCR System (Applied Biosystems, Foster City, CA, USA). Details regarding the qRT-PCR primers are presented in Table S8. Total RNA was extracted from the mesocarp and seed at the five developmental stages using RNAiso Plus Reagent (TaKaRa Bio Inc., Kusatsu, Japan) based on the manufacturer’s protocol, then treated with RNase-free DNase I (New England Biolabs, Ipswich, MA, USA) to eliminate all contaminating DNA. The resulting RNA was applied for first strand synthesis by the PrimeScriptRT reagent Kit with gDNA Eraser (TaKaRa Bio Inc.). The concentration of cDNA was determined and diluted to 12.5 ng/µL. PCR was performed using QuantStudio7 Flex Real Time PCR System (Applied Biosystems, Foster City, CA, USA).The 20-µL reaction volumes comprised 2 µL cDNA, 10 µL SYBR *Premix Ex* Taq™ II (TliRNaseH Plus) (TaKaRa Bio Inc.), 1.0 µL each 10 µM primer, and 6 µL distilled water. The PCR program was as follows: 95 °C for 30 s; 40 cycles of 95 °C for 5 s, melting temperature of each primer for 30 s. The *PaActin7* gene was used as an endogenous control for normalizing data and 2^−ΔCT^ method was used for PCR data analysis. For each sample, the qRT-PCR analysis involved three biological replicates and two technical replicates.

### 4.6. SMRT Sequencing

Poly-T oligo-attached magnetic beads were used to purify mRNA from the total RNA extracted from mesocarp and seed samples collected at each analyzed developmental stage. The mRNA from all five developmental stages was combined to serve as the template to synthesize cDNA with the SMARTer PCR cDNA Synthesis Kit (Clontech, Mountain View, CA, USA). After a PCR amplification, quality control check, and purification, full-length cDNA fragments were acquired according to the BluePippin Size Selection System protocol, ultimately resulting in the construction of a cDNA library (1–6 kb). Selected full-length cDNA sequences were ligated to the SMRT bell hairpin loop. The concentration of the cDNA library was then determined with the Qubit 2.0 fluorometer, whereas the quality of the cDNA library was assessed with the 2100 Bioanalyzer (Agilent). Finally, one SMRT cell each was sequenced respectively with the PacBio RSII system (Pacific Biosciences, Menlo Park, CA, USA) for avocado mesocarp and seed.

### 4.7. Quality Filtering and Correction of PacBio Long-Reads

Raw reads were processed into error-corrected reads of insert (ROIs) using an isoform sequencing pipeline, with minimum full pass = 0.00 and minimum predicted accuracy = 0.80. Next, full-length, non-chimeric transcripts were detected by searching for the poly-A tail signal and the 5′ and 3′ cDNA primer sequences in the ROIs. Iterative clustering for error correction was used to obtain high-quality consensus isoforms, which were then polished with Quiver. The low-quality full-length transcript isoforms were corrected based on Illumina short-reads with the default setting of the Proovread program. High-quality and corrected low-quality transcript isoforms were confirmed as nonredundant with the CD-HIT (version 1) (http://weizhongli-lab.org/cd-hit/).

### 4.8. Analysis of Alpha- and Beta-Carotenes by HPLC

Avocado mesocarp and seed extracts were prepared as previously described [58], with minor modifications. Briefly, fresh avocado mesocarp and seed samples were separately ground in a mortar containing liquid nitrogen. Samples (approximately 2 g) were added to centrifuge tubes, after which they were treated with 4 mL acetone and homogenized (intermediate speed) for 1.5 min at 4 °C. The supernatant was then transferred to a new centrifuge tube, and the extraction of the residue was repeated twice. The extracts were mixed with 5 mL methanolic potassium hydroxide (15%, *w*/*v*), and then saponified for 2 h in the presence of nitrogen. A 3-mL aliquot of the mixture was diluted with 1 mL 10% sodium chloride and then added to a 2-mL solution comprising methylene chloride and water. The supernatant was washed three times with water, evaporated to dryness in the presence of nitrogen, and reconstituted in methanol/methyl tert-butyl ether (85:15). The subsequent HPLC analysis of carotenoids was completed with the 1290 HPLC system (Agilent, Santa Clara, CA, USA) and a YMC carotenoid C30 column (250 × 4.6 mm, 5 μm; Waters, Santa Clara, CA, USA) analyzed at 445 nm. The HPLC mobile phase consisted of methanol/water (96:4, *v*/*v*) and tert-butyl ether at a flow rate of 1.0 mL/min and the column temperature was maintained at 30 °C. The alpha- and beta-carotenes were identified by comparing the retention times of the peaks with those of commercial standards purchased from Sigma-Aldrich (Shanghai, China). Carotenoid contents were quantified based on external calibration curves (*R*^2^ ≥ 0.999). Alpha- and beta-carotene contents were expressed as microgram per gram of fresh weight (μg/g FW). Samples were analyzed with three biological replicates and two technical replicates.

## 5. Conclusions

This study provides a comprehensive overview of the NGST transcriptomes of the avocado mesocarp and seed at five developmental stages. NGST and SMRT transcriptomes results implied that the gene dosage variation and the associated changes in gene expression of most carotenoid biosynthetic-related genes might contribute to the higher carotenoid pathway flux in the avocado mesocarp than in the seed, and accelerate the carotenoid accumulation. The metabolite (alpha- and beta-carotene) profiling via HPLC in the avocado mesocarp and seed during five developmental stages in this study validated the results of our NGST and SMRT transcriptome profiling. Our study results provide new insights into the carotenoid contents and the molecular mechanisms underlying carotenoid accumulation in avocado.

## Figures and Tables

**Figure 2:**
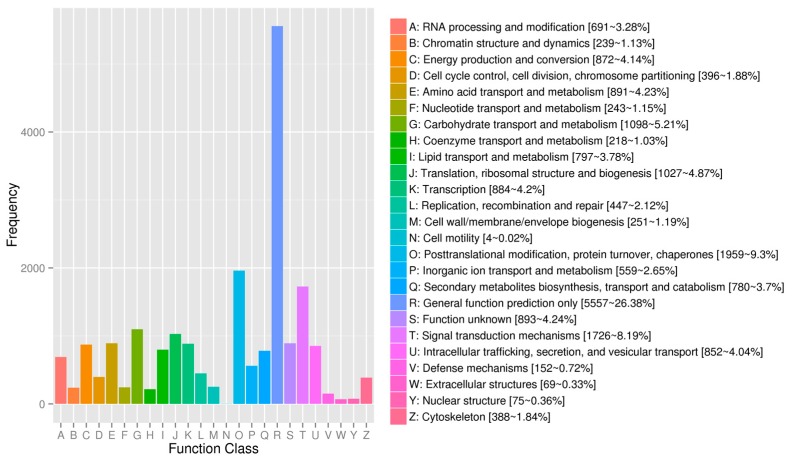
Eukaryotic Orthologous Groups classification of the assembled unigenes.

**Table 1:** Unigenes related to carotenoid biosynthesis.

Gene Name	Functional Protein Name	Enzyme Commission Number	Unigene ID
*PaPSY*	15-cis-phytoene synthase	2.5.1.32	c103350.graph_c0, c113873.graph_c5
*PaPDS*	Phytoene desaturase	1.3.5.5	c103201.graph_c0, c104826.graph_c4
*PaZ-ISO*	15-cis-*ζ*-carotene isomerase	5.2.1.12	c109620.graph_c1
*PaZDS*	*ζ*-carotene desaturase	1.3.5.6	c108741.graph_c1, c115069.graph_c3
*PaCRTISO*	Carotenoid isomerase	5.2.1.13	c108133.graph_c1
*PaLCY-E*	Lycopene *ε*-cyclase	5.5.1.18	c117627.graph_c3
*PaLCY-B*	Lycopene *β*-cyclase	5.5.1.19	c92930.graph_c0, c110018.graph_c0
*PaCYP97A*	P450 *β*-ring carotene hydroxylase	1.14.13.129	c106779.graph_c0
*PaCYP97C*	P450 *ε*-ring carotene hydroxylase	1.14.99.45	c110544.graph_c0
*PaZEP*	Zeaxanthin epoxidase	1.14.15.21	c109893.graph_c0, c116714.graph_c5
*PaNSY*	Neoxanthin synthase	5.3.99.9	c106233.graph_c1, c92501.graph_c0

## Supplementary Materials

The following are available online at https://www.mdpi.com/1422-0067/20/17/4117/s1. Table S1. Phenotypes of avocado cultivar ‘Hass’ at 75, 110, 145, 180, and 215 days after full bloom during the fruit developmental stage; Table S2. Transcriptomic data for 30 avocado samples; Table S3. Gene ontology annotations for the assembled avocado unigenes; Table S4. Enriched Kyoto Encyclopedia of Genes and Genomes pathways among the assembled avocado unigenes; Table S5. Data for the differentially expressed genes in various cDNA libraries for developing mesocarps and seeds; Table S6. Analysis of differential expression and annotation of unigenes related to the carotenoid biosynthetic pathway; Table S7. PacBio library and sequencing results in avocado mesocarp and seed; Table S8. Details regarding qRT-PCR primers; Figure S1. The photos of the tested avcoado samples per fruit developmental stage. Figure S2. Transcript and unigene length distributions; Figure S3. Chromatogram of *α*-carotene and *β*-carotene extracted from 215 DAFB mesocarp and seed of avocado ‘Hass’.
