# Supplementary material for: Transcriptome Profiling Provides Insight into the Genes in Carotenoid Biosynthesis during the Mesocarp and Seed Developmental Stages of Avocado (Persea americana)
Source: Int J Mol Sci. 2019 Aug 23;20(17):4117. doi: 10.3390/ijms20174117 (PMC6747375; doi:10.3390/ijms20174117)

**Figure S3.** Chromatogram of *α*-carotene and *β*-carotene extracted from 215 DAFB mesocarp and seed of avocado ‘Hass’.


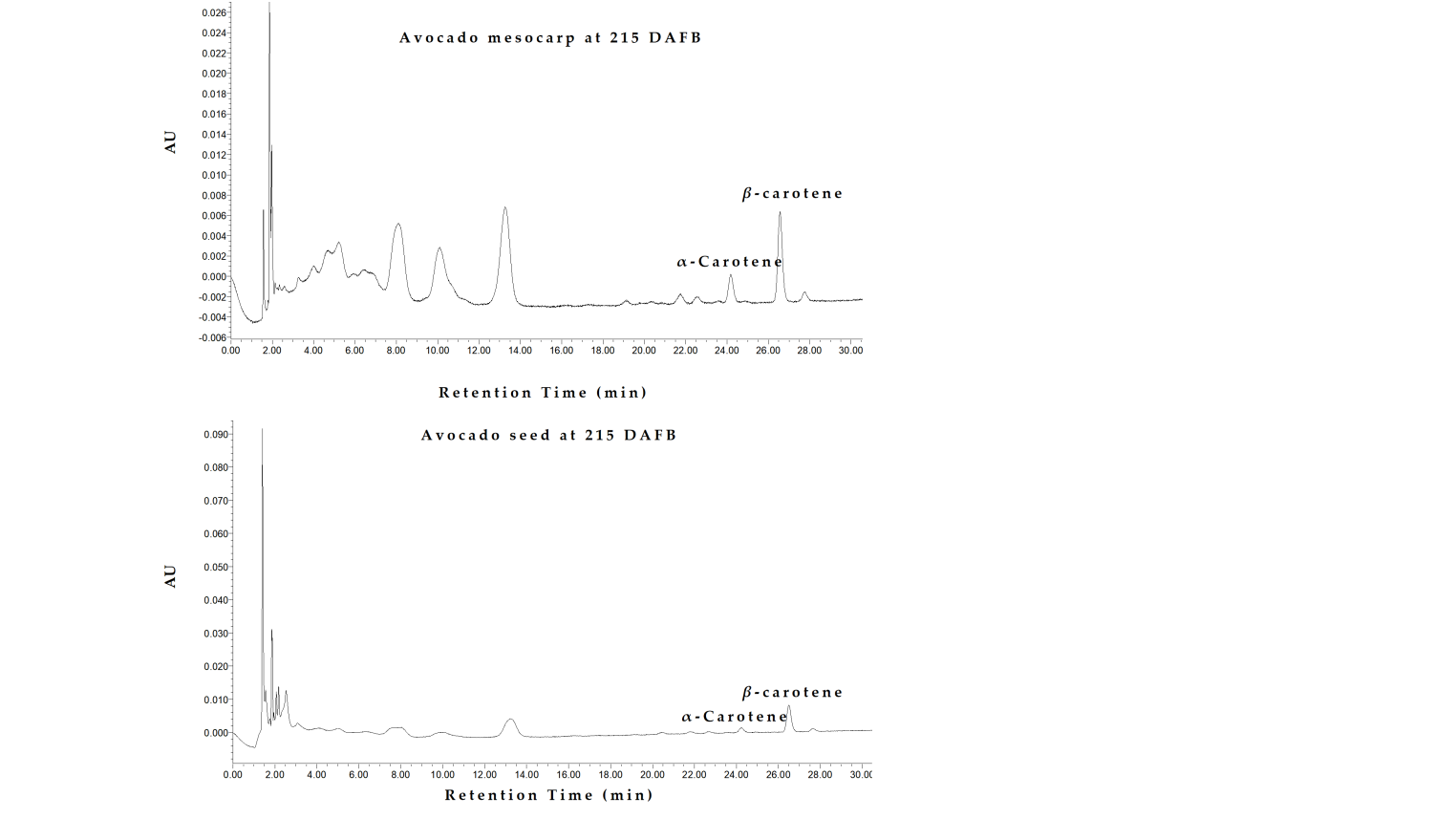

Supplement: Supplementary file 1 [file ijms-20-04117-s001.zip › Supplementary files/Figure S3. Chromatogram of a┴-carotene and a┬-carotene extracted from 215 DAFB mesocarp and seed of avocado í«Hassí».docx]
