# Supplementary material for: Transcriptome Profiling Provides Insight into the Genes in Carotenoid Biosynthesis during the Mesocarp and Seed Developmental Stages of Avocado (Persea americana)
Source: Int J Mol Sci. 2019 Aug 23;20(17):4117. doi: 10.3390/ijms20174117 (PMC6747375; doi:10.3390/ijms20174117)
Supplement: Supplementary file 1 [file ijms-20-04117-s001.zip › Supplementary files/Table S1. Phenotypes of avocado cultivar í«Hassí» at 75, 110, 145, 180, and 215 days after full bloom during the fruit developmental stage.docx]

**Table S1**. Phenotypes of avocado cultivar ‘Hass’ at 75, 110, 145, 180, and 215 days after full bloom during the fruit developmental stage.

| Stage | 75 DAFB | 110 DAFB | 145 DAFB | 180 DAFB | 215 DAFB |
| --- | --- | --- | --- | --- | --- |
| Mesocarp  weight (g) | 21.45 ± 1.39 | 32.15 ± 2.59 | 67.01 ± 4.02 | 96.95 ± 2.76 | 114.74 ± 3.24 |
| Seed  weight (g) | 1.96 ± 0.07 | 3.39 ± 0.21 | 6.67 ± 0.29 | 10.24 ± 0.76 | 11.81 ± 1.07 |
| Fruit  Length (mm) | 49.89 ± 2.07 | 61.49 ± 0.73 | 73.25 ± 1.77 | 77.89 ± 2.12 | 83.07 ± 1.77 |
| Fruit  width (mm) | 39.26 ± 0.67 | 44.73 ± 1.92 | 53.98 ± 1.28 | 58.20 ± 2.39 | 62.42 ± 2.71 |
