# Supplementary figures and images for: Transcriptome Profiling Provides Insight into the Genes in Carotenoid Biosynthesis during the Mesocarp and Seed Developmental Stages of Avocado (Persea americana)
Source: Int J Mol Sci. 2019 Aug 23;20(17):4117. doi: 10.3390/ijms20174117 (PMC6747375; doi:10.3390/ijms20174117)

**Figure S1.** The photos of the tested avcoado samples per fruit developmental stage.


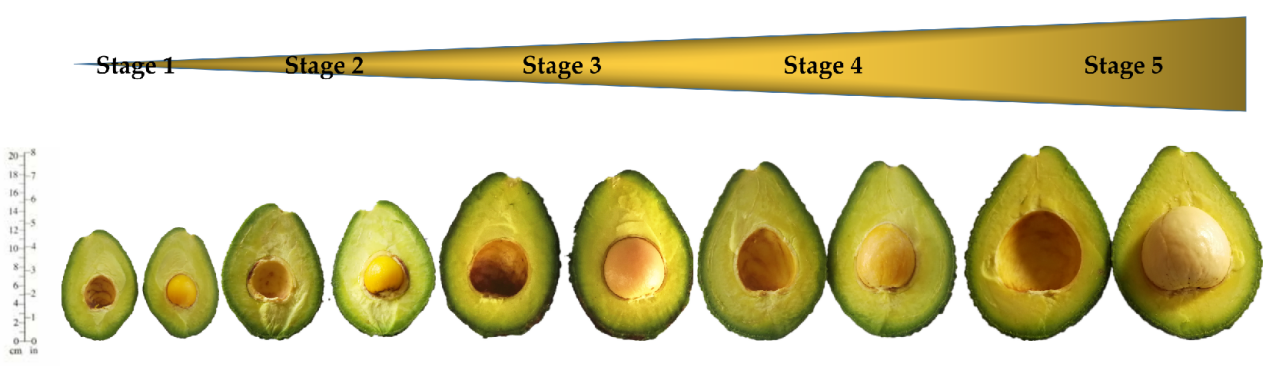

Supplement: Supplementary file 1 [file ijms-20-04117-s001.zip › Supplementary files/Figure S1. The photos of the tested avcoado samples per fruit developmental stage.docx]
